# Notes From the Field: A Voice-Activated Video Communication System for Nurses to Communicate With Inpatients With COVID-19

**DOI:** 10.2196/31342

**Published:** 2022-03-28

**Authors:** Mimi Dunn, Adam Landman, Jennifer Cartright, Anne Bane, Anne Brogan, Caroline Coy, Haipeng Zhang

**Affiliations:** 1 Brigham Digital Innovation Hub Brigham and Women's Hospital Mass General Brigham Boston, MA United States; 2 Brigham and Women's Hospital Mass General Brigham Boston, MA United States; 3 Informatics and Medication Safety Brigham and Women's Hospital Mass General Brigham Boston, MA United States; 4 Nursing Informatics Team Brigham and Women's Hospital Boston, MA United States; 5 Analytics, Planning, Strategy and Improvement Brigham and Women’s Hospital Mass General Brigham Boston, MA United States

**Keywords:** Internet of Things, IoT, voice assistant, telehealth, hospital systems, COVID-19, nurses, nursing, public health, virtual care

## Abstract

With the relaxing of telehealth regulations through the Health Insurance Portability and Accountability Act (HIPAA) waiver notification for Telehealth Remote Communications during the COVID-19 Nationwide Public Health Emergency, our organization had the opportunity to pilot an innovative virtual care solution using a modified consumer-grade voice-activated video communication system (Amazon Echo Show 8) within one inpatient COVID-19 unit. In this brief report, we describe our experiences with implementing the system and general feedback from clinicians, and discuss areas for future development required to enable future scaling of this solution. Our pilot demonstrates the feasibility of deploying a consumer-grade voice assistant device in COVID-19 patient rooms. We found the devices engaging due to the voice technologies and Alexa functionalities for both clinician and patient entertainment. To enable future deployment at scale, enhancements to the Echo Show and data analytics will need to be further explored.

## Introduction

The COVID-19 pandemic has resulted in the unprecedented adoption of virtual care tools [1-3]. Ideally, virtualizing patient encounters in the inpatient setting can preserve the sense of human contact and increase capacity for human connection with patients while making interactions feel less rushed [4]. Additionally, inpatient virtual care tools were needed to both reduce use of personal protective equipment (PPE), especially when supplies were constrained, and reduce in-person contact and risk of transmission. Despite the numerous barriers to telemedicine, such as educating staff, cost, reimbursement, access to broadband, and patient digital literacy, telemedicine has flourished during the pandemic, accelerating implementation timelines that may have been much longer without such a catalyst [5].

In April 2020, Mass General Brigham Health System deployed video intercom communication system (VICS) developed in-house that allowed clinical staff to connect over video to a securely configured tablet inside a patient room [4]. This telehealth solution was implemented to reduce PPE usage and maintain human connection at the bedside. Although quite successful, there were some limitations to VICS—for example, clinical staff required training to use the technology and it was not a hands-free solution, which was an infection control barrier. Solutions in the consumer space, such as Amazon Echo Show (Amazon.com Inc) devices, addressed the hands-free issue and were familiar to staff already but were historically unable to be used in the health care space.

With the relaxing of telehealth regulations through the Health Insurance Portability and Accountability Act (HIPAA) waiver notification for Telehealth Remote Communications during the COVID-19 Nationwide Public Health Emergency [3], the opportunity to explore additional innovative virtual care solutions emerged, including solutions leveraging consumer-grade systems in the clinical setting. One health system found that “the use of consumer products sourced from local vendors is a viable solution for telemedicine systems focusing on speed, reducing costs, and ease of deployment” [6]. In this brief report, we describe our experiences implementing a voice-activated video communication system for clinicians to communicate with inpatients with COVID-19 using consumer-grade hardware (Amazon Echo Shows).

## Methods

### Overview

This pilot project was conducted at Brigham and Women’s Hospital (BWH), a 736-bed, urban academic quaternary care hospital that is a founding member of the Mass General Brigham health care system in Boston, Massachusetts. For the duration of the pilot from September 21, 2020, to November 9, 2020, BWH had a daily average COVID-19 census of 15 patients. In conjunction with nursing leadership, we selected one unit with primarily inpatients with COVID-19 (COVID-19 Special Pathogens Unit) to conduct the pilot project. Selection criteria included a unit that would consistently treat patients with COVID-19 and one that would be adequately equipped to house the pilot devices.

BWH had previously deployed the VICS platform using tablets to improve communication between staff and patients while reducing use of PPE and physical contact time for health care providers treating patients with COVID-19. The configuration of the VICS tablet is locked and has an auto-answer feature enabled so nurses can monitor patients without disturbing them and engage in high-quality 2-way conversations whenever needed without the patient having to take any action.

We used a modified 2-way video communication device (Amazon Echo Show 8) configured to allow drop-in video calls to the patient room to help the nurse conserve PPE by communicating with the patient without having to enter the room numerous times per day. In partnership with a third-party vendor (Aiva Health), a fleet management software system was implemented to enable drop-ins, manage accounts, and provide additional security measures across multiple Echo Show devices. In a similar manner, the Echo Show devices were programmed to allow drop-ins only from the nurse to the patient room. This drop-in function is an instant live connection from a staff device to a patient device, allowing the clinician to see and speak with the patient and the patient to see and speak with the clinician. Patients are unable to initiate an outbound audio or video call; to contact nurses, they would still need to use the nurse call button in their room.

### Amazon Echo Show Device Deployment

A total of 6 patient rooms were included in the pilot. We matched each patient room Echo Show device with a nurse station Echo Show device for a total of 12 devices in the pilot. The Echo Show devices replaced the VICS devices in the 6 patient rooms. The nurse devices were set up at the nurse stations outside each of the rooms (Multimedia Appendix 1). The patient devices were placed on a shelf attached to an intravenous (IV) stand in the patient room (Multimedia Appendix 2). All Echo Show devices were named based on the building name (“Shapiro”) and the assigned number of the device. A new Wi-Fi network was used specifically for the Echo Show devices during this pilot to not interfere with clinical care and as a security measure.

We conducted a live training of the Echo Show device with unit staff prior to the go-live date. Along with the live training, tip sheets were provided for both the nurses and the patients. The tip sheet created for the nurses provided an overview, how-to instructions, important notes, and support guidance (Multimedia Appendix 3). Nurses could drop in to a patient room using voice activation by saying, “Alexa, drop in to Shapiro Echo 2” or by selecting “Shapiro Echo Show” on the touch screen of the Echo Show device.

Patients in the pilot rooms were made aware of the pilot via a patient notification sheet affixed to the Echo Show device in the room (Multimedia Appendix 4). A user guide was also created to explain to patients how to operate the Echo Show device in their room (Multimedia Appendix 5).

We did not encourage patients to touch the Echo Show device. Patients were welcome to explore Alexa functionalities—such as asking everyday questions (eg, weather, news, sports) and listening to music—that did not require any personal account information, although we did not market this feature for the pilot.

Periodic retraining occurred during onboarding of new staff and throughout the duration of the pilot. We collected nurse feedback through regular rounding on the units. The nursing team and technology team feedback was then synthesized and collated into this report.

### Technical Infrastructure and Security

Amazon has a proprietary infrastructure for IP-based audio and video calls that uses the Session Initiation Protocol. The session is initiated and established entirely on Amazon’s network and the 2-way audio/video communication is peer-to-peer between Echo Show devices on the same network, which is end-to-end encrypted and does not go through Amazon’s cloud.

Additional security measures were put in place to ensure Echo Show devices met the security standards of our institution including (1) implementing a fleet management system that automatically deleted all data that originated from the Echo Show devices and (2) configuring the Echo Show devices to connect to a separate Wi-Fi network specifically set up for this pilot to avoid using clinical networks.

## Results

Through rounding on the pilot inpatient unit and compiling comments and emails, we gained valuable feedback on the voice-activated video communication system. The nurses found the Echo Show devices easy to use and the privacy controls were well received. They also enjoyed the additional available features on the Echo Show devices that the VICS system did not have.

## Discussion

### Overview

In addition to the ease of use for video communications using the Alexa drop-in feature, the most positive feedback received was regarding the voice assistant (Alexa) functionality, which was not a feature we advertised. One nurse described how her patient listened to the radio and programming on the Echo using the Alexa functionality and, being from a different country, loved it as it had content she knew and enjoyed. Likewise, nurses enjoyed using the Echo Show at their workstation to listen to the radio using the Alexa functionality, increasing their satisfaction with the system.

### Challenges and Opportunities to Improve Future Voice-Activated Video Communication System Enterprise Deployment

During the pilot, there was interest from nursing leadership to deploy these devices in another building following a COVID-19 outbreak. However, this was not feasible due to several challenges with the current consumer version of the Echo Show device (Table 1).

The Echo Show devices support Wi-Fi Protected Access 2 (WPA2) encryption, which only requires a single preshared key to connect to a network. Learning this single preshared key could lead to a system compromise. Future versions of the Echo Show device can address this by enabling access to WPA2 Enterprise, which requires a unique username and password and a preinstall unique encryption key, thereby providing additional security and enhancing ease of scalability [7].

One of the barriers to implementation was the lack of an official enterprise fleet management solution for the Echo Show devices. A third-party vendor, Aiva Health, created a custom fleet management solution that provided limited enterprise support that was not native to the device. This resulted in the setup of each device becoming a time-consuming activity, taking 1-2 days, as each Echo Show device needed to be configured and tested individually and then brought online by the Amazon and Aiva Health teams with the drop-in feature turned on. If this pilot were to be expanded, additional dedicated team members would be required to manage the implementation.

Due to the HIPAA waiver for telehealth remote communications, this Echo Show device could only be used in COVID-19 patient rooms and had to be removed from the room and unplugged when a patient with COVID-19 was discharged. This made it very difficult for staff to track the devices. No Echo Show devices were lost during the pilot program, but a fleet management solution that included some type of asset tracking system would have improved the experience.

Echo Show device mounting and placement in the rooms and the fixed camera on the device led to additional challenges. The nurses felt that the placement of the Echo Show device on the IV pole was not secure and, depending on the placement of the IV pole, the camera viewing angle from the device could be suboptimal. This led to situations where the Echo Show device was not always facing the patient when the nurse dropped in.

Lastly, there were no built-in data analytics in the Echo Show devices (eg, number of drop-ins, length of drop-in, and number of times voice activation was used), so there was no official way to track device usage. This would have been helpful in understanding the function and usage of the Echo Show device from a quantitative perspective and for measuring the efficacy of this device and comparing it more effectively with usage of the VICS system.

The barriers that previously limited enterprise scaling of voice assistant systems like Amazon Echo Shows are beginning to soften. Third-party vendors, such as Aiva, provide health care–specific fleet management solutions for Alexa devices.

**Table 1:** Summary of challenges in implementing a voice-activated video communication system and potential solutions.

Challenge	Potential solution
The devices only support Wi-Fi Protected Access 2 (WPA2) networks	Updating future devices to support Extensible Authentication Protocol-Transport Layer Security (EAP-TLS) authentication
Many nurses were accustomed to using the existing VICS^a^ solution so they were less interested in adopting the Echo Show device	Provide more live trainings of Echo Show device or completely replace VICS solution with Echo Show device
Device placement on IV^b^ pole was suboptimal as it was not sturdy and was not always facing the patient	Creation of custom mounts for the Echo Show device that can affix to the IV pole and pivot
Lack of data analytics made it difficult to understand usage or efficacy of pilot	Ability to extract and summarize key metrics by device, unit, and user, such as number of drop-ins, length of drop-in, and number of times the voice activation was used

^a^VICS: video intercom communication system.

^b^IV: intravenous.

Further work needs to be done on future devices to support wireless network configurations such as WPA2 Enterprise before these devices can truly be considered for “out of the box” enterprise scaling. Currently, without WPA2 Enterprise support, deploying these devices requires close consideration of how to create a safe and secure connectivity plan. For this pilot, we addressed this through the creation of a stand-alone Wi-Fi network specifically for the Echo Shows. We are hopeful that support for these standards will be adopted in future iterations of the Echo Show hardware.

One of the limitations of this paper is that this is a feasibility pilot in a single unit; more formal research needs to be conducted to help understand and inform further implementation and use of voice-activated video communication systems. Further, we did not formally solicit direct patient feedback on the use of the device. Patient experience is an important area to explore in subsequent research. Finally, this implementation was possible under the HIPAA waiver for telehealth remote communications—additional privacy and security review may be necessary for broader health care use in the future [8].

### Conclusion

Overall, this pilot demonstrates the feasibility of deploying a consumer-grade voice assistant device in COVID-19 patient rooms. Although there are a variety of technologies that can be used to deliver similar 2-way video communication, we found the Echo Show device engaging; it differentiates itself due to the voice technologies and Alexa functionalities for both clinician and patient entertainment [9]. To enable future deployments at scale, security and privacy enhancements to the Echo Show and data analytics will need to be further explored.

## Appendix

Multimedia Appendix 1 Echo Show device at nurse station.

Multimedia Appendix 2 Echo Show device in patient room.

Multimedia Appendix 3 Echo Show nurse instructions.

Multimedia Appendix 4 Patient notification sheet regarding use of Echo Show device.

Multimedia Appendix 5 Echo Show user guide for patients.

## Abbreviations

BWH: Brigham and Women’s Hospital
EAP-TLS: Extensible Authentication Protocol-Transport Layer Security
HIPAA: Health Insurance Portability and Accountability Act
IV: intravenous
PPE: personal protective equipment
VICS: video intercom communication system
WPA2: Wi-Fi Protected Access 2
