# Supplementary figures and images for: Notes From the Field: A Voice-Activated Video Communication System for Nurses to Communicate With Inpatients With COVID-19
Source: JMIR Form Res. 2022 Mar 28;6(3):e31342. doi: 10.2196/31342 (PMC8963263; doi:10.2196/31342)

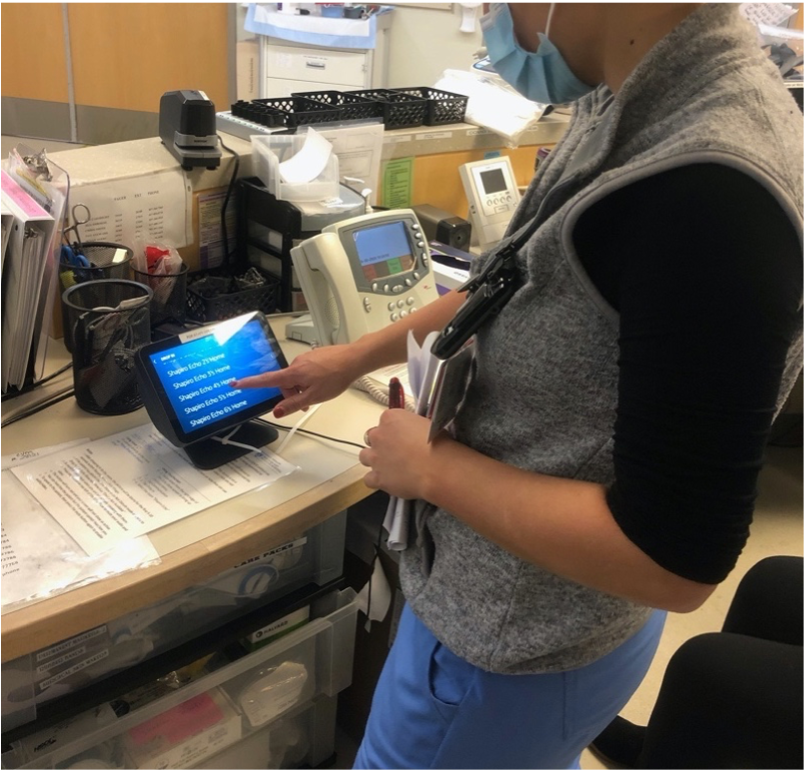

Supplement: Multimedia Appendix 1 [file formative_v6i3e31342_app1.png]

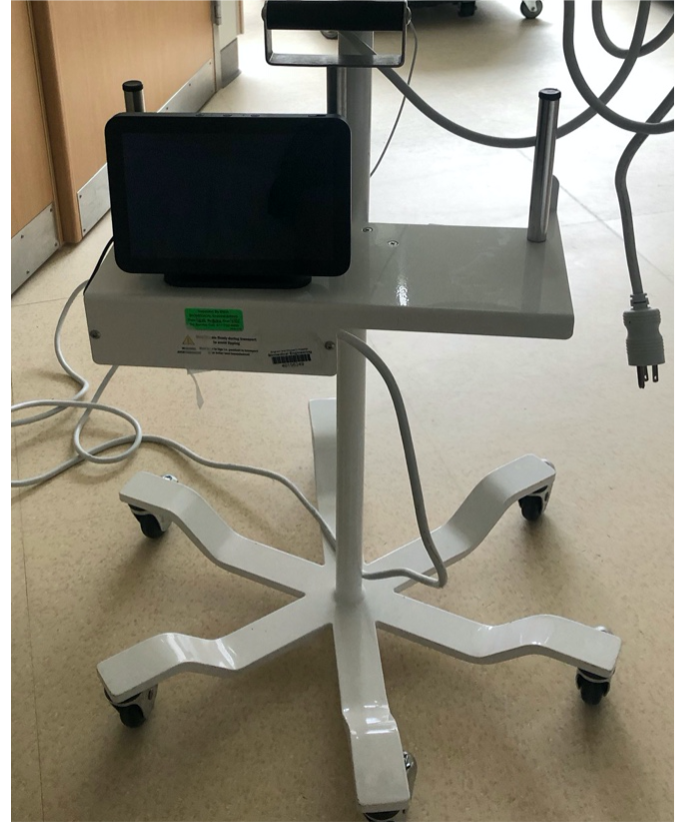

Supplement: Multimedia Appendix 2 [file formative_v6i3e31342_app2.png]

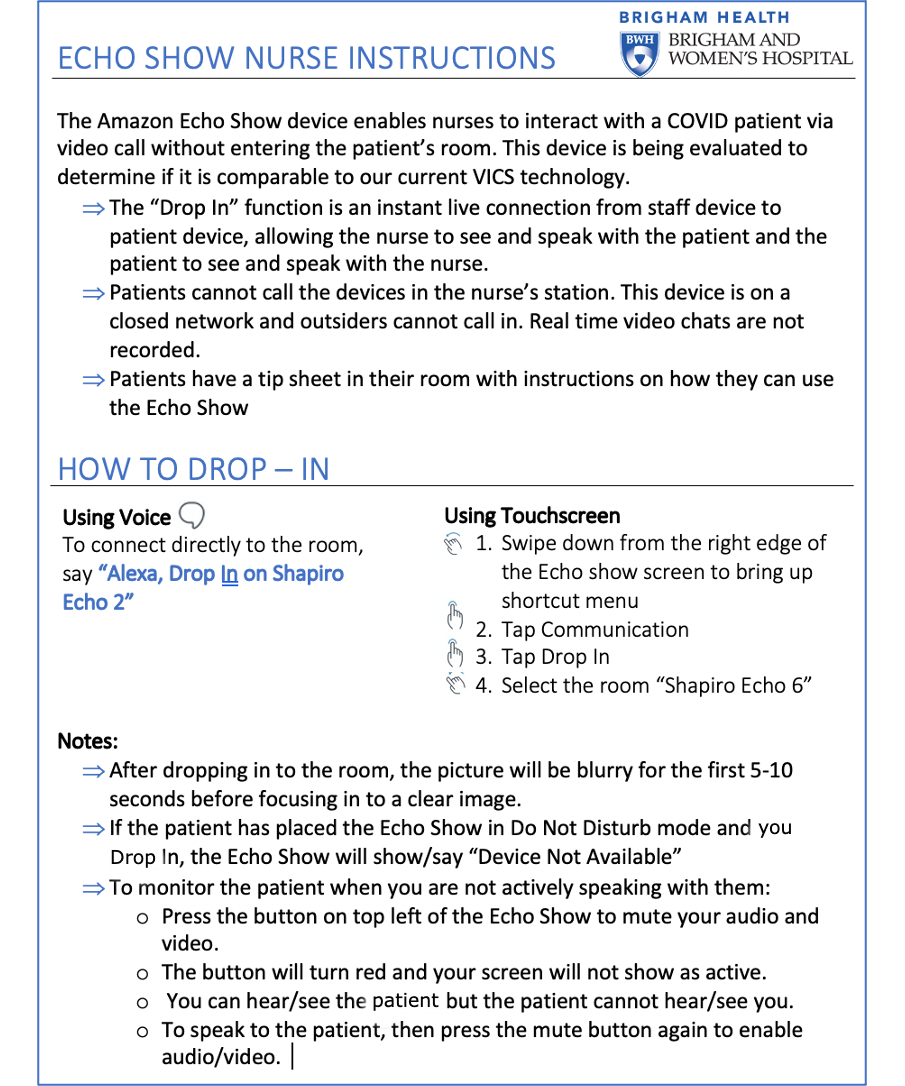

Supplement: Multimedia Appendix 3 [file formative_v6i3e31342_app3.png]

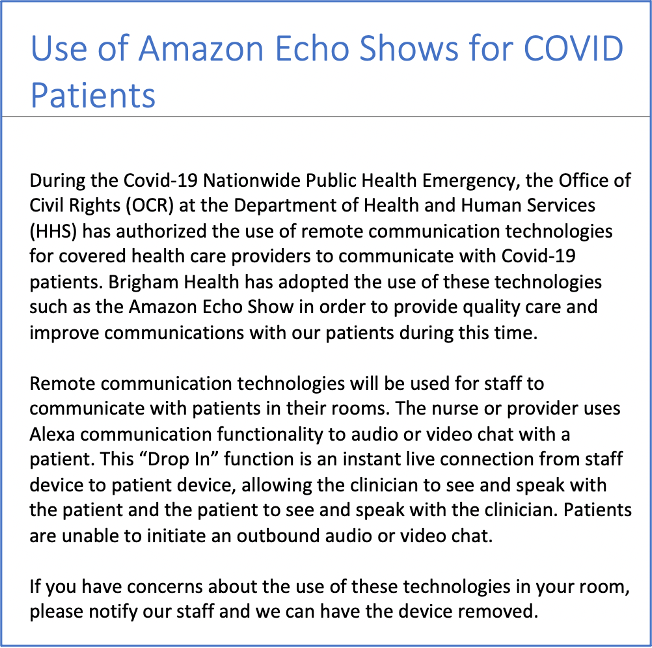

Supplement: Multimedia Appendix 4 [file formative_v6i3e31342_app4.png]

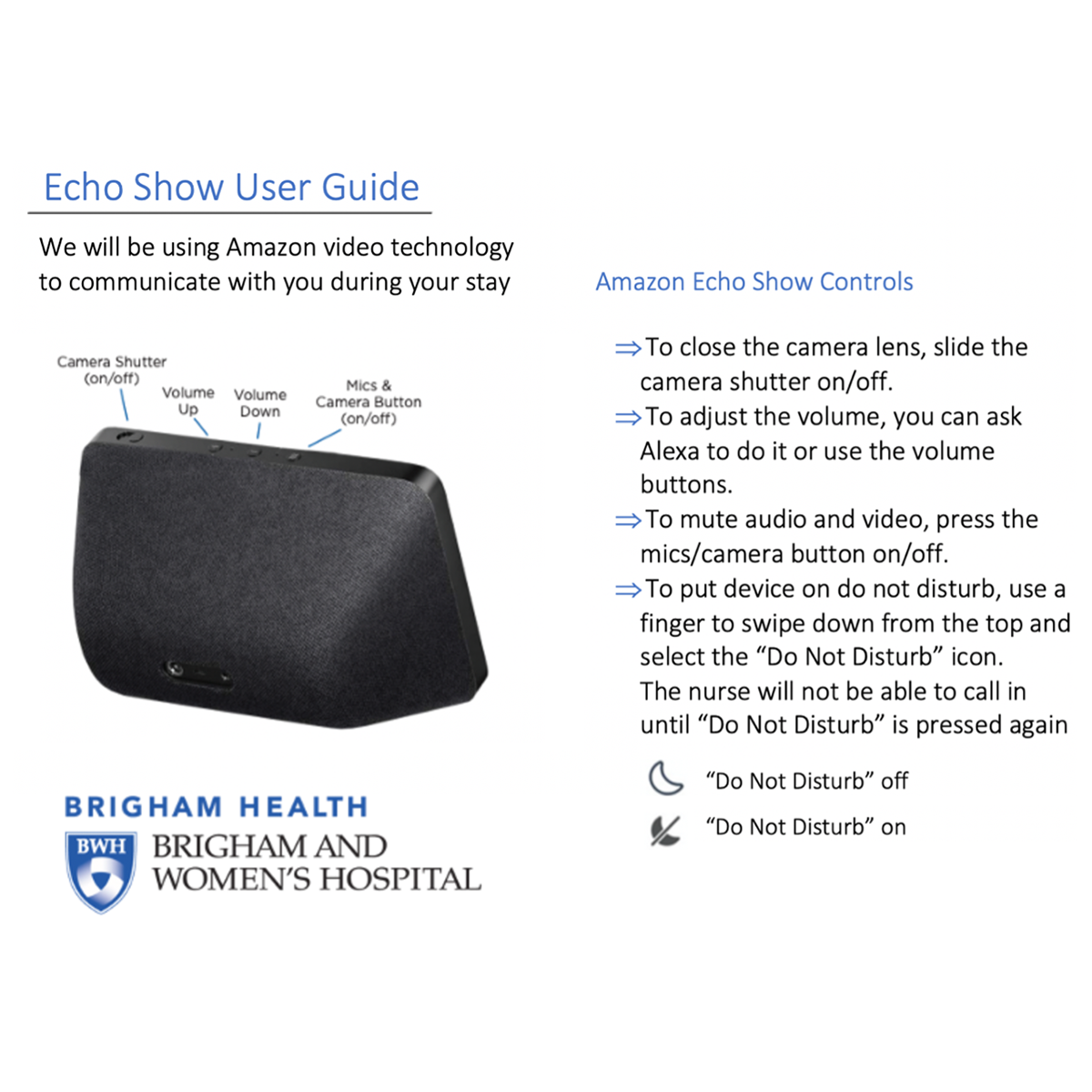

Supplement: Multimedia Appendix 5 [file formative_v6i3e31342_app5.png]
